# Cardiac regenerative capacity: an evolutionary afterthought?

**DOI:** 10.1007/s00018-021-03831-9

**Published:** 2021-05-05

**Authors:** Phong D. Nguyen, Dennis E. M. de Bakker, Jeroen Bakkers

**Affiliations:** 1 Hubrecht Institute-KNAW and University Medical Center Utrecht, Utrecht, Netherlands; 2 Department of Pediatric Cardiology, Division of Pediatrics, University Medical Center Utrecht, Utrecht, Netherlands

**Keywords:** Regeneration, Repair, Evolution, Scar, Extracellular matrix, Inflammatory response, Cardiomyocyte, Proliferation

## Abstract

Cardiac regeneration is the outcome of the highly regulated interplay of multiple processes, including the inflammatory response, cardiomyocyte dedifferentiation and proliferation, neovascularization and extracellular matrix turnover. Species-specific traits affect these injury-induced processes, resulting in a wide variety of cardiac regenerative potential between species. Indeed, while mammals are generally considered poor regenerators, certain amphibian and fish species like the zebrafish display robust regenerative capacity post heart injury. The species-specific traits underlying these differential injury responses are poorly understood. In this review, we will compare the injury induced processes of the mammalian and zebrafish heart, describing where these processes overlap and diverge. Additionally, by examining multiple species across the animal kingdom, we will highlight particular traits that either positively or negatively affect heart regeneration. Last, we will discuss the possibility of overcoming regeneration-limiting traits to induce heart regeneration in mammals.

## Introduction

Regeneration is an injury induced process that can be considered as a combination of multiple synergistic processes that act not only to limit the injury, but also generate new cells to replace the loss of tissue. This regenerative response varies widely within the animal kingdom and can be viewed at multiple biological levels ranging from regeneration of a whole body part, a specific structure or organ, a tissue and to an individual cell [1]. When viewing organ regeneration, in particular the heart, there is considerable variation [2]. Mammalian hearts for example typically lack a regenerative response upon injury, and instead form a permanent scar. Other species, including the fish species Medaka (*Oryzias latipes*) [3, 4] and cave-dwelling *Astyanax mexicanus* [5], show a similar limitation when it comes to cardiac regenerative capacity. In contrast, a wide range of species have been shown to contain robust cardiac regenerative capacity, including the giant danio (*Devario aequipinnatus*) [6], goldfish (*Carassius auratus*) [7], newts (*Notophthalmus viridescens*) [8, 9], Mexican Axolotl (*Ambystoma mexicanum*) [10–12] and surface-dwelling *Astyanax mexicanus* [5]. However, the most commonly used animal to study cardiac regeneration is the zebrafish (*Danio rerio*), which was first reported in 2002 to contain a robust regenerative capacity from amputation of ventricular tissue [13]. Following this seminal study, there have been a plethora of studies examining the cellular and molecular mechanisms contributing to the regenerative response.

Interestingly, cardiac regenerative capacity does not only differ among species, or even sub-populations as is the case with *Astyanax mexicanus*, but also between genetically identical organisms at different life stages. Indeed, several mammalian species have been reported to have a short time-window after birth where they retain regenerative capacity of the heart. For example, neonatal mice can regenerate their heart from injury either via amputation [14] or myocardial infarction (MI) [15] during the first 7 days post birth, with some indication that the regenerative window is restricted to the first 2 days post birth [16]. The neonatal pig also displays this phenomenon whereby its regenerative window lies within their first 2 days post birth [17, 18]. Additionally, there have been clinical reports of human newborns recovering from a MI to various degrees [19, 20].

In this review, we will describe the heterogenous distribution of cardiac regenerative capacity between species. In doing so, we hope to shed light on the processes and traits which cumulatively allow for successful heart regeneration. To achieve this goal, we will first describe processes occurring in mammalian heart repair and zebrafish heart regeneration, indicating where the two process overlap or diverge. Next, we will broaden our scope to include additional species, identifying traits that either positively or negatively affect heart regeneration. Lastly, we will discuss whether these differential processes and traits can be overcome to allow for heart regeneration in mammals.

### Repair vs regeneration: differences between poor and robust regenerators

The repair response following heart injury is shared between zebrafish and mammals. This includes processes involving regulation of cellular stress, the immune system and extracellular matrix deposition that limit the deleterious nature of the injury and secures immediate survival. There are some differences in this repair response when comparing between zebrafish and mammals. Meanwhile these differences become more apparent as time progresses, whereby non-regenerating hearts continue to form a mature and permanent scar while regenerating hearts enter a new phase to replace the lost tissue. Here, we will highlight the shared and differential processes between heart repair and regeneration.

### Extracellular matrix (ECM) deposition

While the adult mammalian heart does not regenerate, it is quite efficient at repair. Indeed, patient fatality from the direct effects of a MI (e.g., heart rupture) is rare. Instead, patients often succumb to mortality from the long-term effects such as cardiomyocyte (CM) hypertrophy, chamber dilation and ultimately heart failure. One factor that contributes to the prevention of heart rupture and direct lethality post MI is the highly coordinated deposition of a dynamic extracellular matrix (ECM) network [21]. This reparative process can be divided into three distinct phases: The Inflammation, Proliferative and Maturation phase [21]. During the inflammation phase, necrotic tissue as well as the native ECM is broken down through the activity of matrix metalloproteases (MMPs) [22, 23]. Simultaneously, increased permeability of vessels bordering the injured area allows for the influx of fibrinogen, forming a provisional fibrin-based matrix network [24, 25]. Through the proliferation phase, which is marked by the proliferation of (myo)fibroblasts, the fibrin-based network is gradually replaced with fibronectin and collagenous type-III filaments secreted by myofibroblasts and macrophages [26–28]. Finally, in the maturation phase, the fibrinous collagen type-III network will be replaced by collagen type-I filaments, which are highly cross-linked and provide robust structural integrity and therefore increasing scar stiffness [29, 30]. Although these ECM dynamics ensure the immediate survival and short-term integrity of heart morphology, the formation of a fully matured, permanent scar leads to adverse effects later in life [31, 32].

ECM deposition during zebrafish heart regeneration also involves the formation of a provisional fibrinous network and consequent replacement by collagenous filaments similar to that seen during mammalian heart repair [33, 34]. However, differences arise when addressing the origin of ECM in the regenerative heart. Besides activated fibroblasts, endocardial cells contribute to ECM production during zebrafish heart regeneration [35]. In addition, the source of pro-fibrotic fibroblasts differs between zebrafish and mammalian hearts. Upon injury, resident fibroblasts of the mammalian heart migrate to the injury site, proliferate and differentiate into pro-fibrotic myofibroblasts under the regulation of TGF-β [28]. In contrast, the pro-fibrotic fibroblasts of the regenerating zebrafish heart not only arise from resident fibroblasts but are also formed by trans-differentiation of epicardial cells [36–38]. Like mammalian fibroblasts, zebrafish fibroblasts are activated through TGF-β signalling to acquire a pro-fibrotic signature, referred to as activated fibroblasts [36]. However, as regeneration progresses these fibroblasts are gradually de-activated to prevent excessive fibrosis [38] and the produced ECM is ultimately replaced by new functional myocardium. The regression of the ECM is dependent on MMPs (such as *mmp2* and *mmp14a/b*) [39] cleaving the collagenous network. However, MMPs might have additional functions during the inflammation phase. Indeed at 4 days post cryoinjury, MMPs are highly expressed by vimentin + fibroblasts (including *mmp9* and *mmp13*) [40]. In addition, *mmp2* and *mmp14a/b* have been shown to be expressed during the inflammation phase 3 days post cryoinjury. In contrast, the upregulation of *mmp2* and *mmp14a/b* only occurs at 7dpi in the apical resection model, which lacks necrotic tissue. This suggests that post cryo-injury, MMPs could play a role in clearance of the necrotic tissue during the inflammation phase. Whether differences in MMP expression could underly differential scar regression between the injured zebrafish and mouse hearts, or whether the difference is due to a change in deposition rate, remains to be elucidated. Besides differences in the origin and temporal dynamics of cardiac fibroblasts and fibrosis, differences in ECM composition are also observed between regenerating and non-regenerating hearts. Interestingly in neonatal mice, the ECM component Agrin is highly expressed during the regenerative window and is downregulated thereafter [41]. This enrichment of Agrin expression is important for promoting CM proliferation and injection of this proteoglycan following adult heart injury improves cardiac function [41, 42]. Whether Agrin is expressed during zebrafish heart regeneration has not been reported, however administration of zebrafish ECM into the MI-induced adult mouse hearts facilitated cardiac functional recovery [43]. This was similarly observed with injection of neonatal cardiac ECM [44]. Thus, showing that ECM components can possess a pro-regenerative role.

Taken together, ECM depositions like fibrin and collagen are shared between the repair and regeneration processes, albeit with differences in their establishment and clearance. Like in the injured mammalian heart, collagen I is expressed in the injured zebrafish heart at 14 days post cryo-injury [45]. Differences between zebrafish and mammalian ECM maturation arise after the initial deposition of collagen I. Mammalian hearts maintain the production of collagen I fibres resulting in a stiff permanent scar. In contrast, the zebrafish heart shows a transient de-activation of pro-fibrotic fibroblasts, thereby limiting the amount of deposited fibrosis [38]. In addition, the zebrafish heart dissolves the initially deposited ECM (including the collagen I network) through the expression of MMPs. To which extend the reduced deposition and/or clearance of the deposited collagenous network determines the transient nature of the zebrafish fibrotic response remains unclear. In addition, it remains to be elucidated which specific differences arise between initial scar deposition between the zebrafish and mammalian heart. The differences in ECM scar composition between regenerating and non-regenerating hearts draw parallels to other forms of regeneration. Spinal cord regeneration in zebrafish for example display a transient stiff ECM that is proposed to stabilize the injury and change to a less stiff ECM to facilitate neuron migration into the injury area ([46], and reviewed in [47]). A similar mechanism may happen in cardiac regeneration to allow for CM to enter and repopulate the scar. Indeed, it has been shown that ECM stiffness correlates to the regenerative window in neonatal mice hearts. Whereby an increase in stiffness correlated with the lost regenerative capacity, while reducing stiffness by addition of an inhibitor at a stage when the regenerative window was closed resulted in the maintenance of regenerative competence [16]. Moreover, ECM production is important for regeneration and repair as ablating collagen producing cells from the injured zebrafish heart leads to impaired heart regeneration [38] and ablating myo-fibroblasts from the post-MI murine heart leads to decreased survival [48]. Furthermore, the composition of the ECM strongly influences the outcome of the heart regeneration and repair processes [49, 50].

### Innate immune system

The inflammatory response is a well-orchestrated, complex process that plays an indispensable role during cardiac repair in mammals. The start of the inflammation phase is marked by the recruitment of neutrophils to the infarct area, which secrete pro-inflammatory signals and attract monocytes to infiltrate the infarction. Resident cardiac macrophages have been shown to recruit monocytes originally derived from the spleen. These monocytes will differentiate into macrophages and play multiple roles while occupying the injury site [51, 52]. This review will conveniently define these roles into two phases (M1 and M2), the separation of these states is much more complex and still not well understood. Nevertheless, these macrophages acquire pro-inflammatory properties (M1) [53] and together with neutrophils will secrete various MMPs that allow for the remodelling of the native ECM [54]. At the same time, damaged and dying cells in the infarct site will activate the complement system, marking necrotic cells for degradation and phagocytosis thereby clearing the infarcted area of dead and necrotic cells [53, 55, 56]. Next, during the proliferative phase, apoptotic neutrophils are cleared from the tissue through phagocytosis by the M1 macrophages, which now progress into a new state (also known as M2) that is marked by the secretion of anti-inflammatory signals [57]. During this phase, a collagenous ECM is produced that is rich in fibronectin. Although myofibroblasts are the main contributors to ECM deposition, it is thought that the M2 macrophages play a regulatory role in ECM turnover through the secretion of MMPs and their inhibitors, TIMPs (reviewed in [58]). In addition, M2 macrophages secrete TGF-β ligands, thereby activating the collagen production in myofibroblasts [28, 59]. During the final maturation phase, collagen deposition is halted and the more loosely organized collagen III is replaced with a tightly cross-linked type I collagen. The role of the innate immune system in this part remains largely unexplored. The role of resident cardiac macrophages has also been shown to play an important role in activating angiogenesis and CM proliferation in neonatal cardiac injury [52, 60]. As well as modulating proinflammatory monocyte macrophages following adult cardiac injury [52].

During regeneration in zebrafish, a similar influx of neutrophils and a presence of various sub-populations of macrophages is observed [3, 61, 62]. A distinct Wt1 + macrophage sub-population, which at least partially arise from the hematopoietic niche was identified that displays a pro-regenerative transcriptomic signature [63]. It remains uncertain how the macrophage populations in the zebrafish compare with the mammalian macrophages. Besides the innate immune system, a recent study indicates that the acquired immune system plays an indispensable role during zebrafish heart regeneration and suggests that differences in the adaptive immune system might underlie differences in regenerative capacity [64]. It would be interesting to determine whether these unique macrophage cell states/subtypes found in zebrafish could be manipulated in mammalian macrophages to potentially improve regeneration.

### Revascularization

During a myocardial infarction in patients, a blood clot restricts blood flow of a coronary artery. This limits the transport of nutrients and oxygen to the downstream region of the heart and cause massive ischemic tissue damage. There has been reports suggesting that injury-induced VEGF signalling leads to local neovascularization, which is a process detrimental to prolonged survival after MI [65–67]. Indeed, the initiation of angiogenesis starts as early as the inflammation phase and continues well into the proliferative phase [66, 68, 69]. First, newly formed vessels are hyper-permeable due to the lack pericytes and smooth muscle cells, allowing the infiltration of leukocytes into the ischemic area. Afterwards, under the influence of PDGF, these vessels obtain a mural coat existing of pericytes and smooth muscle cells reducing permeability [70, 71]. Interestingly, supplying murine ischemic hearts with human pericytes reduced vessel permeability and leukocyte infiltration, which led to beneficial effects on cardiac remodelling. This indicates that targeting neovascularization could have beneficial effects post MI [72, 73]. However, as the infarcted area is remodelled into a mature scar largely devoid of living cells, the demand for nutrients and oxygen plummets. Therefore, as the scar matures the newly generated vasculature becomes obsolete and diminishes accordingly [74, 75].

During regeneration, like during repair, the revascularization is of vital importance. Indeed, revascularization as well as lymphangiogenesis of the injury area is important for efficient heart regeneration in zebrafish [76–78]. Both zebrafish and mammalian revascularization depends on VEGF-signalling, which is expressed in the injury area as early as 15 h (zebrafish) and 6 h (rats) post heart damage [65, 76]. Indeed, injury-induced VEGF has been shown detrimental to prolonged survival after MI in mammals [65–67] and blocking angiogenesis through expression of a dominant-negative Vegfaa blocks cardiomyocyte proliferation and heart regeneration in the zebrafish [76]. The biggest difference is therefore not in the initiation, but in the maintenance of the regenerated vessels. Whereas in the non-regenerative hearts the new blood vessels have no function as the scar matures, regenerated tissue retain their vasculature to support the newly formed high nutrient-/oxygen-dependent CMs. Taken together, revascularization plays a vital role during both repair and regeneration, but is only maintained following regeneration.

### Cardiomyocytes

Cardiomyocyte proliferation in mammals is rare and occurs at a very low rate [79–81]. However, this does not increase nor significantly compensate for the millions of CMs that are permanently lost due to myocardial infarction. One strategy for the surviving CMs to counteract the reduced functionality of the heart is by growing in cell size. Although this hypertrophic response is rapid and quite efficient, it is often not sustainable in the long term. Indeed, many patients that suffered a myocardial infarction end up with pathogenic hypertrophy of the heart, ultimately resulting in heart failure [31, 32]. CMs directly adjacent to the ischemic area, also called border zone CMs, respond in an even more dramatic manner. In order for them to survive the adjacent infarction, they partly dedifferentiate towards a more immature state [82, 83]. This occurs through the replacement of a MEF2-driven gene program, defining adult CM cell fate, with a stress-responsive AP-1-driven gene program allowing for survival under ischemic conditions [84]. Indeed, knock-out of one of these border zone stress responsive factors, *nppb*, results in acute death following myocardial infarction in mice [84].

Instead of displaying a hypertrophic response due to cardiac insult in mammals, regenerative species respond with a hyperplastic response. In the zebrafish heart, the CMs that are lost by the injury are replaced by proliferation of existing CMs in the border zone [85, 86]. In doing so, the heart will be restored to its original properties and deposited fibrosis will be replaced by new functional myocardium [13, 34]. Border zone CMs in the regenerating heart also dedifferentiate and activate a stress program indicated by the expression of stress induced genes such as members of the AP-1 complex (i.e., *junba*, *junbb*, *fosab*, *fosl1a*) as well as *nppa* and *nppb*, much like mammalian border zone CMs during heart repair [84, 87]. However, this cellular reprogramming seems much more pronounced because it includes metabolic reprogramming from a fatty acid towards glycolysis dependent ATP production [88, 89], as well as the re-activation of an embryonic gene program [37, 85]. Indeed, a recent study has shown that the transcriptome of zebrafish border zone CMs is more similar to embryonic CMs then to remote myocardial CMs originating from the same hearts [88]. This reversion back to an embryonic state is likely key to unlock their proliferative potential as inhibiting their dedifferentiation, including the induction of glycolysis, prevents CMs from proliferating effectively [86–89].

Taken together, the reparative heart employs a hypertrophic response to deal with the loss of heart functionality, while the regenerative heart instead uses hyperplastic regrowth. Although the hypertrophic response forms an adequate short-term solution, it leads to severe problems in the long term, including complete heart failure. Border zone CM of both the reparative and regenerative heart dedifferentiate and activate a stress response program. However, this response seems to deviate in the regenerative heart, where the dedifferentiation results in the initiation of an embryonic-like gene program and the induction of glycolysis, while in the mammalian BZ many genes with high expression in neonatal CMs are not induced [84]. The induction of the embryonic-like gene program, including the induction of glycolysis, might help explain why zebrafish BZ CMs proliferate while mammalian BZ CMs do not re-enter the cell-cycle. Another explanation for the limited proliferative capacity of mammalian BZ CMs might be due to the intrinsic properties of cardiomyocyte nuclei. While mammalian CMs are mainly polyploid (human) or multinuclear (mice), zebrafish CMs are mononuclear and diploid, which has been shown to be detrimental for efficient proliferation and zebrafish heart regeneration [90–92].

**Fig. 1:**
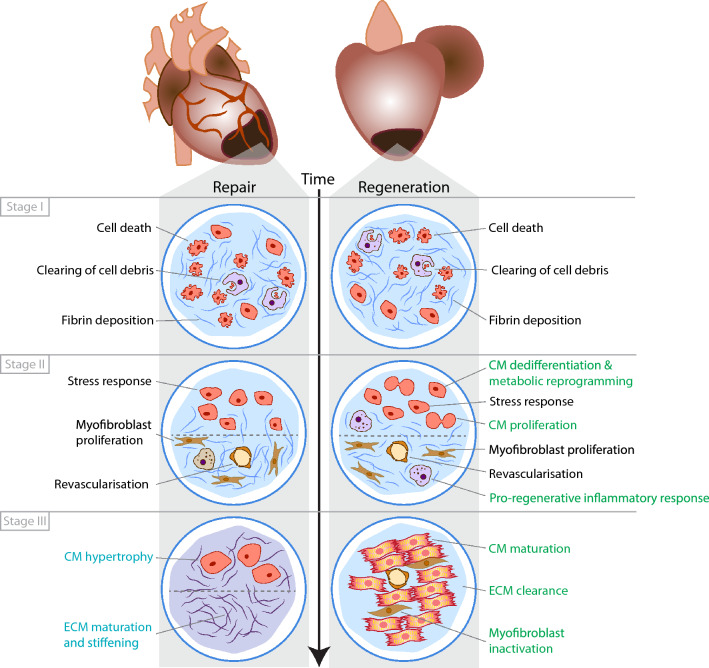
Comparison of repair vs regenerative response following cardiac injury. Schematic summary of the main processes and their response in animals that can either repair or regenerate following cardiac injury. This is also summarized in Table 1

In summary, while the reparative and regenerative response show many similarities, distinct differences are observed as well (Fig. 1 and Table 1). Specifically targeting individual differences might help to stimulate the regenerative response in endogenously reparative species such as mammals.

**Table 1:** Comparing adult mammalian heart repair with zebrafish heart regeneration

Process	Repair	Regeneration
ECM deposition: fibrin	Yes	Yes
ECM deposition: collagen	Yes	Yes
ECM maturation	Yes	No
ECM clearance	No	Yes
Innate immune system: neutrophils	Yes	Yes
Innate immune system: inflammatory macrophages (M1 phase)	Yes	No?
Innate immune system: anti-inflammatory macrophages (M2 phase)	Yes	Yes?
Neovascularization	Yes	Yes
Stress response border zone CMs	Yes	Yes
Reverting back to embryonic state/metabolic reprogramming	No	Yes
CM proliferation	No	Yes
CM hypertrophy	Yes	No

### Regenerative traits: processes that facilitate a positive environment for regeneration

**Fig. 2:**
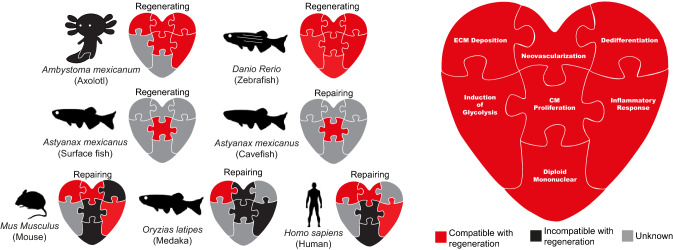
Regenerative traits viewed as pieces of a jigsaw that facilitates cardiac regenerative capacity. Summary of the current literature in regards to a specific regenerative trait and whether it can facilitate normal adult cardiac regeneration in Axolotl, Zebrafish, Human, Medaka, Mouse and Cavefish. Red represents confirmed trait involved in facilitating regeneration. Black represents confirmed trait that is incompatible with regeneration. Grey represents traits in which have not been directly tested. References for these traits are summarized in Table 2

As discussed above, for cardiac regeneration to occur, many independent processes have to be precisely regulated and aligned. Recent studies have indicated the existence of regeneration-specific enhancers, which might have originated from repurposed injury responsive enhancers [93–95]. In fact, the loss of regeneration-specific enhancers might explain the lack of a regenerative response in some species [93]. While positive natural selection might occur on regeneration as a single unit, i.e., by maintaining regeneration-specific enhancer elements, competition for selection will likely also occur on the level of the individual processes constituting regeneration. In other words, environmental changes might force adaptations in a certain trait which is beneficial for the survival of the species, while being incompatible with cardiac regeneration. The second possibility is that a lack of evolutionary pressure (relaxed selection) would lead to the disappearance of cardiac regenerative capacity due to neutral evolution. The evolutionary mechanism leading to limited regenerative capacity is likely to differ between animal species and remains currently unresolved for mammals. In this section of the review, we will discuss the most prominent traits that impact cardiac regeneration. Furthermore, we will provide examples of species that have adapted these regeneration-compatible traits, likely losing the ability to regenerate their hearts in the process (Fig. 2 and Table 2).

**Table 2:** Summary of the known and unknown literature of cardiac regenerative capacity in various species

	*Ambystoma mexicanum* (Axolotl)	*Danio rerio* (Zebrafish)	*Homo sapiens* (Humans)	*Oryzias latipes* (Medaka)	*Mus musculus* (Mouse)	*Astyanax mexicanus* (Cavefish)
ECM deposition	[10, 11]	[33, 34]	[84]	[3, 4]	[84]	[5]
Neovascularization	[10, 11]	[76]	[67]	[3, 4]	[73]	Unknown
Dedifferentiation	[10, 11]	[37, 85]	Unknown	Unknown	[84]	Unknown
Metabolic reprogramming	Unknown	[88, 89]	Unknown	Unknown	Unknown	Unknown
CM proliferation	[10, 11]	[13, 96]	[79]	[3, 4]	[14, 15]	[5]
Inflammatory response	[10, 11]	[63, 97, 98]	[99]	[3]	[100]	[5]
Diploid mononuclear	Unknown	[90]	[101]	Unknown	[81, 102]	Unknown

### Regeneration trait: cellular roles of macrophages

As mentioned above, the innate immune system, in particular the inflammatory response is one of the first responses to an injury. Aside from phagocytosing cellular debris at the injury area, macrophages also play multiple roles in the subsequent cellular responses. For example, they can mediate the fibrotic response and directly contribute to collagen deposition [103], promote neoangiogenesis [3, 52, 100, 104] and initiate CM proliferation [52, 105]. The importance of this cell population becomes apparent when overall depletion or ablation of specific macrophage subsets in zebrafish result in impaired regeneration with a decrease in CM proliferation and increase in scar formation [3, 63, 97, 98, 106].

The importance of macrophages during cardiac regeneration can also be observed in other species. In Medaka fish, heart regeneration does not occur and coincides with a delayed and reduced macrophage recruitment [3]. Meanwhile salamanders (axolotl), which are normally pro-regenerative display an inability to regenerate following macrophage depletion [10]. Likewise, macrophage depletion in neonatal mice following cardiac injury during the regenerative window resulted in an increase of fibrotic scar formation and decrease in cardiac output despite displaying a normal CM proliferation rate typically seen during regeneration [100]. All together these studies point to the positive role of macrophages in creating a permissive environment for regeneration.

The primary role of the innate immune system (and critically the M1 macrophages) is to defend from infection and repair damaged tissue. However, it appears that the M2 phase is an acquired state that plays a key role in the regenerative phase. One role for M2 macrophages is to secrete signals that stop the pro-inflammatory M1 phase through the release of many factors such as TGF-B and IL10 [107, 108]. However, this regenerative phase depends on whether the initial injury persists or not. When there are chronic injury signals, M2 macrophages can instead activate and exacerbate fibrosis [103, 109]. Additionally, comparisons between the transcriptomes of medaka and zebrafish macrophages showed the changes in gene expression profiles were similar, however the medaka profiles were less dynamic and consequently there was a reduced and delayed inflammation following injury. This again suggests that medaka macrophages still possess the ability to facilitate regeneration but the signal is not potent enough to induce this phase. Especially since exogenous activation of Toll-like receptors agonists (which elicits the acceleration of macrophage recruitment and neutrophil clearance) in injured medaka hearts boosted regeneration [3].

Taken together, it appears that medaka have lost the ability to regenerate their hearts through adaptations in their inflammatory response. These adaptations include less dynamic macrophages, delayed neutrophil clearance and delayed macrophage recruitment to the injury site [3]. This dampening of inflammation follows the theory that there is an inverse relationship between organ and limb regenerative capacity and the strength of the inflammatory response within the innate immune system [110–112]. However, it remains unclear why medaka fish have evolved an altered innate immune system. A distinct difference from the regenerative zebrafish is that medaka stay longer in their chorions [113], thereby providing medaka embryos with a safe environment. Potentially, the prolonged protection by the chorion reduced the need for a strong injury response during early development. However, whether this resulted in the evolutionary adaptations seen in the medaka innate immune system, remains unclear.

### Regeneration trait: modifying DNA content—polyploidy and polynucleation

Cells can exist with a varying amount of DNA material in the form of polyploidy (more than two sets of chromosomes per nuclei) and polynucleation (more than one nucleus). This phenomenon arises through the fusion of neighbouring cells or resulting from complete DNA replication without mitosis or cytokinesis (endoreplication) [114]. For example, adult hepatocytes, skeletal muscle, certain cell types in the lung, kidney, pancreas and CMs are typically polyploid [114]. In CMs, ploidy differs between species. CMs of non-mammalian species such as fish, amphibians and reptiles are mainly mononucleated and diploid. While at least 50% of the CMs in mammals such as humans, rodents, bats, livestock and even whales are multinucleated and polyploid [115]. Initially, mammalian CMs are predominately mononucleated and diploid, but this changes after birth. In the mouse heart, CM polyploidization commences during the first week after birth [102, 116], after the loss of the cardiac regenerative potential period [14, 16], while in humans, the persistence of polyploidy is maintained throughout life [101]. It is unclear why humans and mouse whom are both poor regenerators evolved to have different compositions of DNA content in their CMs (multinucleated vs polyploidy, respectively). However, we have grouped these two events into one trait of modifying DNA content since as we will further discuss, the same end result in both contexts appears to modulate cardiac regeneration capacity.

The relation between ploidy and regenerative capacity of the heart was established in a survey of more than 120 genetically defined inbred mouse strains with variation in CMs ploidy. Furthermore, genetic experiments in mice confirmed such a correlation between the percentage of mononucleated CMs and regenerative capacity [91, 92]. In zebrafish, a direct correlation between ploidy and regenerative capacity was also established. The majority of CMs in zebrafish are mononucleated and diploid [90, 117], however increasing the percentage of polyploid CMs impaired regenerative capacity of the heart [90]. While in zebrafish and mice, a correlation between CM mononucleation and regenerative capacity was established, this correlation seems less clear in the porcine heart. In the porcine heart, polyploidization with multinucleation occurs over a 2-month period while loss of heart regeneration potential occurs at P3 [118].

The benefit of polyploidization is not well understood. One idea is that the polyploidy state prevents DNA damage [119] since the polyploidy state in hepatocytes have been shown to play a tumour-suppressive role in the liver [120]. Therefore, the stabilization of the nucleus facilitates the longevity of the organ and this trade-off for regenerative potential may allow cells to survive and maintain function. This would be important in the context of the heart as its function is regular contractions and unstable cells within the heart would compromise this role. Another explanation would be to support organ growth. It is theorized that the increase of polyploidy can support the growth of the cell via increased gene expression and therefore maintain higher cellular activity [121]. In the case of CMs, a larger cell would allow increased metabolic rates for energy production to support the metabolically intensive activity of contractions [122].

Taken together, mammals like mice and humans have adapted the nuclear content of cardiomyocytes, favouring multinucleated or polyploidy nuclei, respectively. While these adaptations might have resulted in distinct benefits such as a reduced risk of DNA-damage and potent regulation of cellular growth, it also limits cardiomyocyte proliferation and therefore heart regeneration.

### Regeneration trait: energy consumption

Endothermal animals are able to self-regulate their body temperature as opposed to ectothermic animals that acquire the temperature of their environment. Thus, far adult heart regeneration capacity has only been reported in ectothermic fish and amphibians and not in endothermic birds or mammals and this may be attributed to difference in energy metabolism as endotherms have a higher resting rate metabolism [123]. This increase in resting rate metabolism is due to elevated aerobic metabolism, which is a very efficient process of energy production. Aerobic metabolism requires oxidative phosphorylation (OXPHOS) that occurs inside mitochondria. When less oxygen is available to cells, they can revert to glycolysis and lactate fermentation for energy metabolism, which generates lactate. While OXPHOS is much more efficient in producing energy as in ATP, it leads to the generation of reactive oxygen species (ROS), which can cause DNA damage. While the adult heart mainly utilizes fatty acids and OXPHOS for the generation of ATP, during mammalian development the foetus grows in an oxygen poor environment and the heart mainly utilizes glucose and glycolysis for energy metabolism. The transition from a low oxygen environment in utero, to an oxygen rich environment after birth coincidences with a shift in energy metabolism of cardiomyocytes from glycolysis towards the oxidation of fatty acids and OXPHOS [124, 125], 126. This also coincides with an increase in ROS production, DNA damage and a strong reduction in cardiomyocyte proliferation [126]. The increase in OXPHOS activity after birth impairs CM proliferation as reducing environmental oxygen after birth, inhibiting fatty acid utilization or promoting ROS scavenging prolongs the proliferative window of cardiomyocytes [126, 127].

Besides the negative effects of OXPHOS and ROS production on CM proliferation, CM require glycolysis and lactate fermentation for efficient proliferation. In the regenerating zebrafish heart proliferating CMs in the border zone shift their energy metabolism from OXPHOS to glycolysis and when this is prevented CM proliferation and regeneration is impaired [88, 89]. Conversely, stimulating glycolysis promotes CM proliferation [89]. The regulation of energy metabolism is highly complex and involves many pathways. Neuregulin 1 (Nrg1) is an agonist for the Epidermal Growth Factor Receptor Tyrosine Kinase family which includes ErbB1, 2, 3 and 4 [128]. There are several indications that in the heart this pathway plays an important role in controlling CM proliferation through regulation of energy metabolism. First, during development, Nrg1/ErbB2 signalling is important for cardiac development as knock out mice for these genes and zebrafish *erbb2* mutants are embryonically lethal as a result of a thinner myocardium due to reduced CM proliferation [129–134]. Second, CM-specific expression of a constitutively active form of ErbB2 (caErbB2) results in cardiomegaly (large hearts) via increased CM proliferation [131]. In addition, when Nrg1 is overexpressed specifically in zebrafish CMs in an absence of injury, the fish also develop cardiomegaly by enhanced CM proliferation [135]. Third, activation of Nrg1/ErbB2 signalling in mouse and zebrafish hearts stimulates glycolysis in embryonic and adult CMs [88, 136]. In the context of heart regeneration, Nrg1 expression is induced in the zebrafish heart upon injury, which again is required for the activation of glycolysis and induction of CM proliferation [88, 135].

Why is a shift from fatty acid oxidation to glycolysis important for CM proliferation? One could argue that since mitochondrial OXPHOS generates ROS which is capable of inducing a DNA damage response and cell cycle arrest [126], 137, it may be better for the proliferative cell to revert to a lower energy producing state to reduce ROS production. Another argument could be that during proliferation, since there is a disassembly of the contractile apparatus [88, 96], the high energy consumption is not needed as compared to a functionally contracting CM and therefore the more inefficient energy producing pathway would suffice. Finally, intermediate metabolites produced by aerobic glycolysis may be converted into precursors for the biosynthesis of amino acids and nucleotides that are essential for cell proliferation and growth [137, 138]. In addition, there have been reports that components of the glycolytic pathway directly interact with cell cycle regulators, which is independent from their catalytic activity, [139, 140] and therefore this pathway may be required to activate the proliferation programme.

The trade-off between regenerative potential and metabolism can also be seen in Mexican cavefish. This teleost species (*Astyanax mexicanus*) is a single species comprising of two populations. A cave-dwelling (Pachón) and surface-dwelling population. About 1.5 million years ago, these two populations diverged due to the changing environment of the Mexican rivers and caves and resulted in the Pachón population losing certain features such as their eyes and pigments [141], and instead acquire new traits such as highly sensitive taste buds and lateral line neurons for navigating in the dark [141]. Interestingly, due to the food scarcity within the caves, the Pachón have adapted by changing its glucose metabolism [142, 143]. In particular, RNA-seq between these fish populations indicate a downregulation of glycolysis-related genes [5]. Moreover, the Pachón population cannot regenerate its heart following injury when compared to their surfacing dwelling counterparts [5]. While yet to be tested, if the surface dwelling fish also require a glycolytic switch in their CMs to facilitate CM proliferation, this would indicate the regeneration trade-off in the Pachón population may be beneficial for other functions that allow this species to survive.

Metabolism and endothermy are highly linked since endotherms have higher metabolic rates compared to ectotherms. The origin of endothermy in birds and mammals is a controversial topic in evolutionary biology. Several hypotheses have been proposed to explain its evolutionary origin of which the aerobic capacity model received most attention [123, 144, 145]. Physiological studies indicate that resting and maximal rate metabolism are linked so that one cannot increase without the other. The aerobic capacity model proposes that an increase in resting rate metabolism supported an increase in maximal rate metabolism to allow sustained muscle activities. High maximal oxygen consumption rates allow sustained workloads by aerobic metabolism, which is beneficial for many activities such as capturing prey or sustained flight. In addition, the increase in resting rate metabolism facilitated the regulation of body temperature. The increase in resting rate metabolism by mitochondrial OXPHOS requires higher oxygen consumption, which may have resulted in adaptations such as a more efficient ventilation system and an increase in blood circulation. Numerous adaptations seen in hearts of mammals and birds, such as separation of the chambers and increased wall thickness, accommodate a more efficient oxygen transportation to all tissues [146]. Therefore, heart regeneration, which depends on the ability of CMs to switch energy metabolism from a very efficient fatty acid oxidation to a more inefficient glucose metabolism by glycolysis and lactate fermentation, may have been a trade-off for an increase in energy demands in endotherms. This may not be restricted to only heart regeneration since a comparable metabolic switch towards glycolysis was also observed during appendage regeneration [147].

The higher availability of oxygen for energy production could in part be due to the aquatic to terrestrial transition which began approximately 500 to 400 million years ago [148]. Some possible reasons for this transition could be due to animals living during the Ordovician–Silurian extinction event, a period of marine O_2_ deprivation [148]. Another reason an increase in atmospheric O_2_ concentration allowed for bigger animals to form and therefore gain a competitive advantage against potential predators [149]. The link between animal size and atmospheric O_2_ concentration is evident as giant animals became extinct when O_2_ concentrations reduced [150, 151]. While it is tempting to suggest that the transition from aquatic to terrestrial environment for vertebrates caused the loss of the regenerative trait, it is possible this trait developed independently of this transition.

### Inducing regenerative traits in species with a poor regenerative capacity

All vertebrates can regenerate to an extent, the difference lies in the capacity of the particular organ. Thus, one could view regeneration as a dormant process in organs that do not regenerate and manipulating this process can reactivate the organ’s ability to proliferate. Following this reasoning, there have been attempts to boost CM proliferation in mammalian hearts. The modulation of both Hippo and ErbB2 signalling and most recently the interaction between these two pathways can robustly induce CM proliferation and regeneration following injury [131, 152–154]. In all cases, when proliferation is induced, a number of cellular and molecular processes are activated that involve some of the above-mentioned regenerative traits. For example, mononucleated CMs preferentially undergo proliferation compared to binucleated CMs [131, 155]. Meanwhile both zebrafish hearts via Nrg1 overexpression and mouse hearts via caErbB2 overexpression switch in metabolism towards glycolysis, which is required for CM proliferation [88]. In addition, ectopic expression of Pkm2, an isoenzyme of the glycolytic pyruvate kinase, in injured mouse hearts induces CM proliferation and restored cardiac function [156]. While the induction of the Nrg1/Erbb2 pathway appears to directly affect certain regeneration associated traits (e.g., metabolic reprogramming), it appears it can completely bypass others (e.g., polyploidy) by focussing on a specific subset of CMs (mononuclear, diploid). Understanding the factors that can overcome the regenerative block by examining interactions between pro-regenerative pathways (e.g., Nrg1/Erbb2 signalling) with the traits and processes affecting regenerative capacity, will help us develop novel methods of inducing CM proliferation and subsequent cardiac regeneration in mammals.

## Concluding remarks

Cardiac regeneration is a multimodal process that requires the precise regulation of numerous processes and cell types. It is unclear what the origin of cardiac regeneration is, but common features such as activation of CM proliferation suggest that it may have a common origin. How cardiac regeneration has been maintained or lost in specific animals is under extensive debate [1]. The loss of cardiac regenerative capacity can result from adaptations in any of the traits and processes described in this review, including the innnate immune system, nuclear organization and metabolism. The heterogeneous distribution of cardiac regenerative capacity throughout the animal kingdom might therefore be a direct consequence of the complexity of the regeneration process. Here, we have summarized several examples of species that might have lost cardiac regenerative capacity through adaptations in distinctly different traits. Therefore, we might consider cardiac regenerative capacity as “an evolutionary afterthought”, only when no other traits take precedence, cardiac regeneration becomes an evolutionary priority to maintain, and this could be explained by either the pleiotropy or adaptive hypothesis [1]. In the pleiotropy scenario, regeneration would be retained because it is developmentally tightly controlled to other adaptations. Cavefish for example changed their metabolism to adapt to the scarce food availability and this metabolomic state is also linked to CM proliferation. The adaptive hypothesis predicts that a trait is actively being maintained during selection and regeneration would be viewed to being negatively selected over repair. In general, the relative body size in poor vs. robust cardiac regenerators is anticorrelated, thus the energy and mechanical requirements for the heart to properly function and sustain the body is much higher in bigger and poor regenerators. Therefore, the need to quickly repair the injury and maintain heart function is being selected over the more slower regeneration process. The energy required to regenerate could also be expensive since the CMs themselves undergo dramatic changes to proliferate and mature, compared to a more simpler repair process. Thus, repair may be favoured in non-cardiac regenerators to divert energy expenditure to other energy intensive processes/organs. Alternatively, a phylogenetic inertia scenario could describe regeneration as this theory predicts the traits are being maintained for historical reasons and confer no selective advantage or disadvantage, therefore this trait was not yet been eliminated from the collection of traits an animal has acquired for survival [1]. Comparative studies between species allow us to identify differences and similarities between species and better understanding of the traits and processes underlying or limiting cardiac regeneration in these different species could potentially help overcome the limited regenerative capacity in mammalian hearts.
